# Overcoming limitations in current measures of drug response may enable AI-driven precision oncology

**DOI:** 10.1038/s41698-024-00583-0

**Published:** 2024-04-24

**Authors:** Katja Ovchinnikova, Jannis Born, Panagiotis Chouvardas, Marianna Rapsomaniki, Marianna Kruithof-de Julio

**Affiliations:** 1 Urology Research Laboratory, Department for BioMedical Research, University of Bern, Bern, Switzerland; 2 IBM Research Europe, Zurich, Switzerland; 3 Department of Urology, Inselspital, Bern University Hospital, University of Bern, Bern, Switzerland

**Keywords:** Cancer, Cancer

## Abstract

Machine learning (ML) models of drug sensitivity prediction are becoming increasingly popular in precision oncology. Here, we identify a fundamental limitation in standard measures of drug sensitivity that hinders the development of personalized prediction models – they focus on absolute effects but do not capture relative differences between cancer subtypes. Our work suggests that using z-scored drug response measures mitigates these limitations and leads to meaningful predictions, opening the door for sophisticated ML precision oncology models.

Precision oncology aims at data-driven identification of personalized treatments for cancer patients. Drug sensitivity tested on cell lines or organoids has shown great potential for predicting therapy success^1,2^. In this approach, cancer cell lines or organoids are exposed to a large collection of anti-cancer compounds at different concentrations in vitro. The cancer cell survival rate is then used to derive measures of drug efficacy, such as the half maximal inhibitory concentration (IC_50_) or the area under the dose-response curve (AUC)^3^. With the ever-increasing availability of large data resources containing drug sensitivity measurements and paired omic profiles across hundreds of cell lines^4–7^, machine learning (ML) models have emerged as a promising approach towards predicting drug response^8–13^. ML models for drug response prediction typically integrate omic data from cancer cell lines with drug profiles to predict drug sensitivity, as measured by IC_50_ or AUC^13,14^.

Several studies have so far addressed open questions on how to train ML models for drug response prediction. Notably, Sharifi-Noghabi et al.^14^ carried out a systematic study on the comparative performance of several ML models when trained and tested on the most popular cell line datasets to predict different measures of drug response. In agreement with previously reported striking discordances between two large pharmacogenomic datasets^15^, namely CGP^6^ and CCLE^5^, cross-domain generalization issues that question the application of ML models in clinically relevant tasks have been reported^14^. The use of IC_50_ as a proxy of therapeutic efficacy has also been considerably debated^14–17^, as IC_50_ indicates potency and not necessarily clinical outcomes. Additionally, IC_50_ is highly dependent on the cell division rate^17^ and the overall drug toxicity^18^. The latter suggests that when comparing drugs by IC_50_ values alone, more toxic drugs would be unnecessarily prioritized, hindering personalized predictions. To address these issues, alternative scores have been proposed. The AUC, a metric that is independent of the dose and captures the cumulative effect of the drug, is less related to mere potency and was reported to better explain systematic variation in cancer drug response^16^. Other alternative scores include the drug relevance score (ratio of the drug’s IC_50_ to its maximum therapeutic dosage) that approximates the therapeutic index used in drug development^18^, the normalized version of AUC (i.e., the ratio of the drug’s AUC to the maximum area for the concentration range)^19^, the normalized growth rate inhibition (GR)^17^ that compares growth rates in the presence and absence of drug, accounting for confounding effects of division rate, the activity area (AA)^5^ that reflects both drug efficacy and potency, and the drug sensitivity scoring (DSS)^20^ that integrates multiple dose-response relationships in cancer and control cells. Still, the vast majority of ML models for drug response prediction are trained to predict IC_50_ or AUC^13,14^.

Motivated by the above, here we investigate how standard measures of drug response impact ML models of precision oncology. We used GDSC, the largest resource that forms the basis of most current ML models, containing IC_50_ values of hundreds of anticancer drugs in thousands of cancer cell lines^4^. We first observed that IC_50_ drug response profiles were very highly correlated even between cell lines of distinct origins (example of a bladder carcinoma and a glioma cell line shown in Fig. 1a). We extended this analysis to all pairs of cell lines in GDSC and found that this effect was omnipresent (i.e., consistent across all cancer subtypes; Fig. 1b, Supplementary Fig. 1), local (i.e., pronounced within cell lines of the same subtype; Supplementary Fig. 1), and robust (i.e., consistent across all drug pathways, see Supplementary Fig. 2). Following the same process for two distinct and widely used cancer cell line datasets, namely the Cancer Cell Line Encyclopedia (CCLE)^5^ and the Cancer Therapeutics Response Portal (CTRP)^21–23^, corroborated our observations (Fig. 1b). This surprising finding indicates that drug response, as measured by IC_50_, is largely dependent on the drug and not the cell line it was tested on, suggesting that predicting drug response based on IC_50_ is a trivial task. Viewing GDSC through the lens of the *drug*, previous work has observed high correlation across drugs reported as a “general level of drug sensitivity” by Geeleher et al.^24^ and “general response across drugs” by White et al.^25^, in part explained by multi-drug resistance. Conversely, our analysis views drug sensitivity prediction through the lens of the *cell* by using drug response to compute pairwise cell line similarity. When repeating our analysis using the drug relevance score^18^ (IC_50_/max concentration) and the AUC, we confirmed our observation, even if pairwise correlation coefficients were slightly decreased (Fig. 1b, left). Finally, we followed the same process using PCPL, a pancreatic cancer patient derived organoid (PDO) library with associated genomic, transcriptomic, and therapeutic profiling^19^. Using the normalized AUC still leads to the same problem of elevated correlation coefficients as observed in GDSC (Fig. 1b, right). Together, our findings point to a fundamental issue when using these measures for personalized drug response prediction from cell line or organoid data: drug response is heavily affected by the inherent potency or toxicity of each drug *independently* of the cell line it was tested on.

**Fig. 1:**
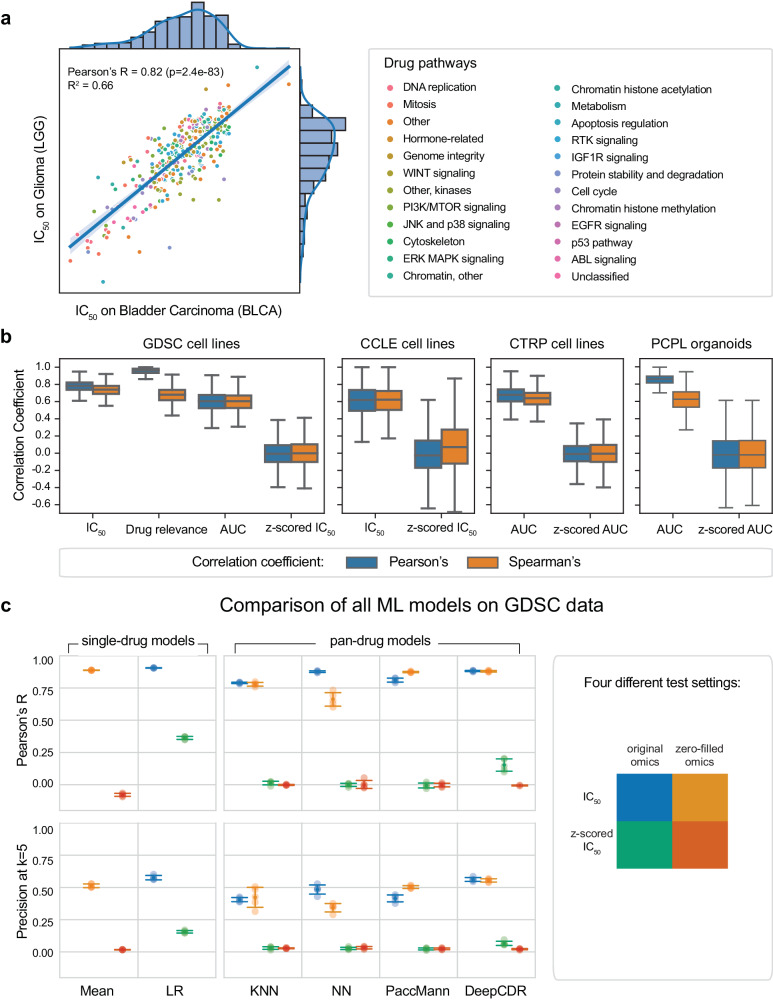
Different measures of drug response on cell line datasets. **a** A scatterplot of IC_50_ values of all drugs tested on two cell lines (Bladder Carcinoma and Glioma) indicates a high correlation between the corresponding IC_50_ drug response profiles. **b** Boxplots of pairwise Pearson and Spearman correlation coefficients for different measures of drug response (e.g., IC_50_, AUC), computed for each pair of GDSC, CCLE and CTRP cell lines and PCPL organoids. For the GDSC boxplots, the correlation coefficient was computed across all tested drugs shared across a cell line pair, which made up on average 81% ± 19% of all drugs. In all box plots, center line corresponds to the median, box limits to the upper and lower quartiles and whiskers to 1.5x interquartile range. **c** Performance of different ML models of drug sensitivity prediction using the Pearson correlation coefficient between observed and predicted drug response measures (top) and the Precision at *k* = 5 score (bottom) across all cell lines. The dots and errorbars represent five independent cross-validation folds, and corresponding standard deviation, respectively. KNN k-nearest neighbor, NN neural network, Mean mean baseline, LR linear regression. The pan-drug models were tested using both original and z-scored IC_50_ values, with original or zero-filled omics feature vectors, resulting in a total of four test setting combinations, as indicated in different colors in the legend. The mean baseline model was evaluated without omics data and the linear regression model was evaluated with omics data only.

We next explored how this issue affects the performance of ML drug sensitivity prediction models. We first implemented single-drug models, including a mean baseline model that assigns to each test sample the mean drug response of that drug from the train set, and a linear regression (LR) model on selected genes (Methods). We compared these baseline models with four pan-drug ML models: (i) a bimodal k-Nearest Neighbor (kNN) model^26^, (ii) a fully connected neural network (baseline NN), (iii) PaccMann, a multi-modal attention-based neural-network model^27^ and (iv) DeepCDR, a hybrid graph convolutional network^28^ (Methods). To mitigate the reported limitations, we applied a z-score normalization for all IC_50_/AUC values separately for each drug, thus removing the drug-specific bias and dominance of toxic compounds. Indeed, as expected, correlation coefficients computed for z-scored IC_50_/AUC values are now reduced and centered around zero (Fig. 1b). To test the contribution of omics in the prediction, we tested all models with normal or zero-filled omics feature vectors, resulting in a total of four test settings (Methods and Fig. 1c, legend). All settings were tested in a cross-validation fashion using both actual and z-scored IC_50_ values. Our results clearly demonstrate that even the least sophisticated models can accurately predict IC_50_ values (Fig. 1c, top). Model performance is not affected in the zero-filled omic setting, suggesting that indeed, the predictions solely rely on drug profiles and are indifferent to molecular properties of the cell lines (Fig. 1c, top). Strikingly, the mean baseline model that simply outputs the average IC_50_ without considering the omic profiles achieves comparable results to more sophisticated ML models. Predicting the z-scored IC_50_ values appears to be a much more challenging task, with all pan-drug ML models largely failing in all settings (Supplementary Data 1–3). Repeating the same process using the precision at *k* score (Methods, Fig. 1c, bottom) and a rank-based loss function^18^ (Supplementary Fig. 3) is insufficient to mitigate the issue, indicating that our results are consistent across performance assessment scores. This can be explained by considering that z-scoring removes the per-drug systematic variation from the IC_50_ measurements, and thus predicting the z-scored IC_50_ values implies learning to predict how cell lines respond to drugs relative to an average cell line. Overall, we conclude that, although ML models can accurately predict IC_50_, these predictions are not in reality personalized but rather merely driven by drug features that are universal across all cancer types. In other words, established drug response metrics promote learning absolute effects of drugs while neglecting relative differences between cell lines thus counteracting a vision of precision oncology where subtleties in biological signatures drive treatment decisions. This can be mitigated through the usage of response metrics that emphasizes relative differences such as the proposed z-scored IC_50_.

To better understand this effect, we looked deeper into the PCPL pancreatic organoid dataset and visualized drug response of the top three drugs in terms of AUC (Fig. 2a, top) and z-scored AUC (Fig. 2a, bottom) across all organoids (results on all tested drugs are given in Supplementary Fig. 4). We also predicted drug rankings across all tested organoids based on its AUC and z-scored AUC value (Fig. 2b top and bottom, respectively). A first striking observation is that Bortezomib has the lowest AUC and is thus the top-ranking drug in almost all tested organoids. Disulfuram follows closely and ranks second in almost 80% of all tested organoids, followed by SN-38. Conversely, z-scored AUC values appear to be organoid-specific, with different drugs being highly effective for different organoids (Fig. 2a, b, bottom), suggesting they can potentially be used for personalized drug recommendations. To assess this, we evaluated all previous models for predicting both the AUC and z-scored AUC in a full and zero-filled omics setting (Methods). We first employed a zero-shot inference setting, i.e., we trained pan-drug models on the cell line data (GDSC) and tested them on the PCPL data (Fig. 2c, left). All pan-drug models reached satisfactory performance when predicting the AUC without additional re-training. However, the models again relied only on drug profiles: zero-filling the omics data did not diminish performance, the simplistic mean baseline model outperformed all ML models, and all models failed in the z-scored setting (Supplementary Data 4–6). The single-drug linear regression model performs slightly worse than the pan-drug models, suggesting that it is highly dependent on the gene selection. We then trained and tested all models on the PCPL dataset in a 10-fold cross validation setting (Fig. 2c, right). As expected, pan-drug models reach higher overall performance, but are again dependent only on the drug profiles. Interestingly, this time the linear regression model achieved accurate predictions in both the AUC and z-scored AUC setting, both in terms of Pearson’s correlation coefficient (Fig. 2c, right) and Precision at k (Supplementary Fig. 5). This encouraging finding suggests that, under appropriate considerations, predicting drug sensitivity from omics data is feasible even with simplistic models.

**Fig. 2:**
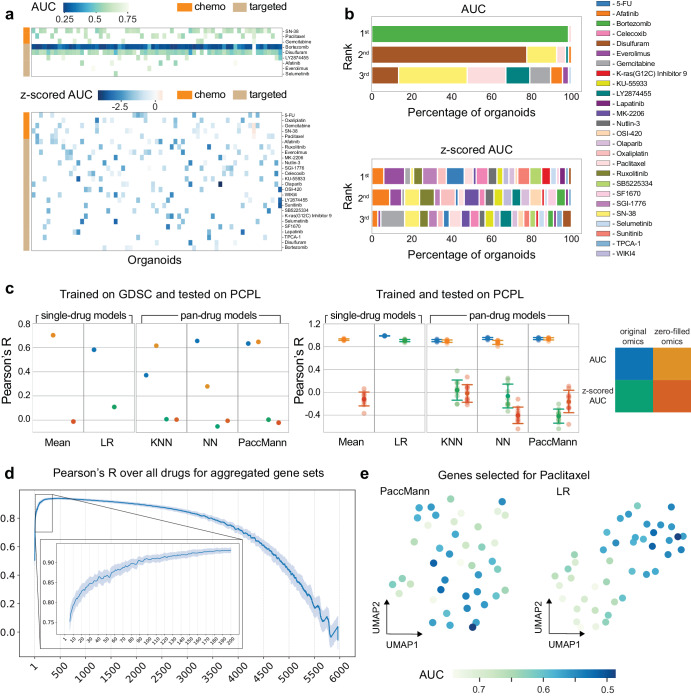
Results on pancreatic organoid data. **a** Heatmap of top three drugs as per AUC score (top) and z-scored AUC score (bottom) for each PCPL organoid (column). Smaller/higher AUC values indicate higher/lower efficacy of the drug in the tested organoid; smaller/higher z-scored values indicate higher/smaller efficacy related to the average efficacy of that drug across all organoids. **b** Stacked bar plots indicating the percentage of PCPL organoids in which each drug ranked 1^st^, 2^nd^, or 3^rd^, as per AUC (top) or z-scored AUC score (bottom). **c** Pearson’s R correlation coefficient between predicted and observed drug response values computed per organoid for each of the evaluated models. All models were either trained on GDSC and tested on the PCPL dataset (left) or trained and tested on the PCPL dataset only (right). In the latter, error bars indicate the standard deviation across 10 cross validation folds. **d** Pearson’s R correlation coefficient between predicted and observed AUC values for varying number of considered genes aggregated over all drugs. **e** UMAP embedding of the RNAseq organoid data using genes selected by PaccMann (left) and the drug-specific gene selection scheme of the linear regression model (right) for Paclitaxel. Here, each dot corresponds to an organoid, and the color of the dot reveals the AUC values of Paclitaxel on that organoid.

Motivated by this, we further investigated genes important for the linear regression predictions. We first removed multicollinearity in RNAseq data, resulting in a total of 6000 linearly independent genes (Methods). By computing the prediction performance in terms of average Pearson’s correlation coefficient for varying number of selected genes (Fig. 2d), we see that the prediction quality is stable when the number of relevant genes varies between 100 and 1000 and reaches a maximum value of around 0.93 for approximately 350 genes on average, a pattern consistent across all tested drugs (Supplementary Fig. 6). Using the genes selected for each drug as features, we created a 2D embedding map using UMAP^29^ and visualized the AUC of a selected drug (Paclitaxel) on that embedding (Fig. 2e). We observe that dataset and drug-specific gene selection allows for a straightforward linear separation of the organoids in terms of their AUC, whereas genes selected based on the literature do not have the same effect. This observation is true for all tested drugs (Supplementary Fig. 7) and explains the high performance of the linear regression model: unlike pan-drug models trained on GDSC, the linear model is specific to the PCPL dataset, which could be an advantage in a disease-specific clinical setting. To further assess the performance of the linear regression model, we created scatterplots of predicted vs. observed z-scored AUC values per drug (Supplementary Fig. 8) and per organoid (Supplementary Fig. 9), which indicated consistent results at an individual drug or organoid level. Together, our results suggest that personalized drug response prediction from omics measurements is indeed feasible and opens the door for more sophisticated ML models to be applied on the same single-drug, z-scored setting.

In conclusion, in this work we expose fundamental issues with the use of common drug response measures that hinder the application of ML models for pan-cancer personalized drug response prediction. Specifically, we show that popular ML pan-drug sensitivity prediction models do not base their predictions on the molecular features and fail to make truly personalized predictions. Although using z-scored IC_50_/AUC values mitigates this issue by reformulating the drug response prediction into prediction of drugs to which a patient responds better or worse than an average patient, training models in a z-scored setting is a challenging task for all pan-cancer ML models. Conversely, we show that even simple models may learn from omics data, performing exceptionally well when trained in a drug-specific manner on a well-defined disease setting. Future efforts in developing personalized drug sensitivity prediction models need to address limitations associated with IC_50_/AUC and consider adopting response metrics with drug-specific transformation schemes, such as z-scoring. A key challenge on the road toward developing improved pan-cancer models is to equip them with inductive biases to promote omics features. This enhancement can facilitate predictions for previously unseen drugs while maintaining the ability to provide personalized treatment recommendations. Finally, the use of organoids to predict responses to cancer drugs represents a significant stride forward in precision medicine, as supported by recent studies^30–33^. The potential to tailor treatments based on individual organoid responses holds considerable promise for improving the efficacy of cancer therapies while minimizing adverse effects. As progress unfolds in this field, predictions of drug responses derived from organoids may become an integral component of personalized cancer treatment strategies.

## Methods

### Datasets

Cell line data were obtained from the Genomics of Drug Sensitivity in Cancer (GDSC) database^4^ (we used the GDSC1 data), the Cancer Cell Line Encyclopedia (CCLE) dataset^5^ and the Cancer Therapeutics Response Portal (CTRP) dataset^21–23^ (details in Data Availability). For GDSC, the drug screen results are available in terms of viability per dosage and were used to obtain IC_50_ and AUC values. All ML models except DeepCDR were evaluated on 953 GDSC cell lines and 283 drugs. Since DeepCDR was evaluated using both transcriptomic and genomic data, we trained and tested it with the original GDSC subset provided in the DeepCDR repository (https://github.com/kimmo1019/DeepCDR) which contained 561 cell lines and 238 drugs. The pancreatic cancer dataset was obtained from the Pancreatic Cancer PDO Library^19^ (PCPL) containing RNA expression data for 43 organoids that were screened against 26 drugs. The drug efficacy was measured and reported in terms of AUC values.

### Computation of correlation coefficients

Let $({x}_{i,j},{x}_{i,k})$ denote the drug responses of drug $i\in [1,n]$ in cell lines $j,k\in [1,m]$ respectively, reported as e.g., the IC_50_, AUC or drug relevance score. Then, the Pearson correlation coefficient *r*_*j,k*_ between cell lines *j*, *k* and across all drugs *n* is computed as:

$${r}_{j,k}=\frac{\mathop{\sum }\limits_{i=1}^{n}\left({x}_{i,j}-\bar{{x}_{j}}\right)\left({x}_{i,k}-\bar{{x}_{k}}\right)}{\sqrt{\mathop{\sum }\limits_{i=1}^{n}{\left({x}_{i,j}-\bar{{x}_{j}}\right)}^{2}\cdot \mathop{\sum }\limits_{i=1}^{n}{\left({x}_{i,k}-\bar{{x}_{k}}\right)}^{2}}}$$

where $\bar{{x}_{i}}$ and $\bar{{x}_{j}}$ correspond to the mean drug response of cell line *j* and *k* respectively, across all drugs. We note that, as it is often the case that not all drugs were tested in all cell lines, only the common drugs between the two cell lines are used for the above computation. Similarly, the Spearman correlation coefficient is computed for each pair of cell lines across all drugs by considering the rank values of *x*.

### Z-score transformations

The z-score transformations of the drug responses *x*_*i,j*_ were computed for each drug across all cell lines it was tested on, or specifically:

$${Z}_{i,j}=\frac{{x}_{i,j}-\bar{{x}_{i}}}{{\sigma }_{i}}$$

where $\bar{{x}_{i}}$, *σ*_*i*_ correspond to the mean and standard deviation of the drug response of drug *i* across all cell lines it was tested on, respectively. For GDSC, we used the precomputed z-scored values as provided in the GDSC data, but we independently validated that indeed they were performed across all cell lines.

### Drug sensitivity prediction from GDSC

#### Pan-drug models

We first tested several pan-drug prediction models that integrate molecular features (genomic or transcriptomic data of cell lines/organoids) with chemical structural properties of drugs and can thus predict drug response for both seen and unseen drugs. In this approach, the whole dataset is employed to train one prediction model. The following models were considered, in increasing complexity: (i) a baseline bimodal **k-Nearest Neighbors (kNN)** model proposed by Born et al.^26^ which predicts drug response of a test sample (cell line or organoid) as the average of the drug responses of the *k* nearest samples in a bimodal similarity space defined using an inversed Tanimoto similarity of Morgan fingerprints as drug-drug distances^34^ and the Euclidean distance for gene expression features. (ii) a **baseline neural network (NN)** consisting of 3 fully connected hidden layers and a linear output unit as used by Prasse et al.^18^ The network takes as input a concatenation of encoded SMILES and gene expression features, and outputs a drug response value. The network is trained using MSE of the real vs. the predicted IC_50_ as a loss function. (iii) **PaccMann**, a multi-modal attention-based neural-network model with state-of-the-art performance in IC_50_ prediction from transcriptomics data^27^. PaccMann relies on SMILES, and uses contextual attention layers to merge information across both modalties. PaccMann incorporates prior knowledge about targets of drugs present in GDSC and protein-protein interactions and reduces the dimensionality of the gene expression data to 2128 genes. Similarly to the baseline NN, PaccMann employs an MSE loss function. We used the PaccMann implementation from https://github.com/PaccMann/paccmann_predictor. In a recent study^18^, PaccMann has been modified to predict ranking of drugs rather than IC_50_ values by replacing the MSE loss with a normalized discounted cumulative gain (NDCG). This modification was shown to outperform the reference models with respect to the drug ranking task. We tested this model with the implementation available from https://github.com/PascalIversen/mlmed_ranking. (iv) **DeepCDR**, a hybrid graph convolutional network consisting of a uniform graph convolutional network (UGCN) representing chemical drug features and multiple subnetworks representing different types of omics profiles^28^. UGCN relies on the adjacent information of atoms in a drug and aggregates the features of neighboring atoms together. The subnetworks generalize over features of cell line omics profiles. The high-level drug and omics features are then concatenated and used as input for a final prediction. DeepCDR utilizes gene expression, genomic mutation, and DNA methylation data and only considers 697 genes from COSMIC Cancer Gene Census (https://cancer.sanger.ac.uk/census). The network is trained using MSE as a loss function. For a fairer comparison of DeepCDR with other models, we initially tested only with transcriptomics data and found that it predicted the same drug response for each cell line. We thus decided to use DeepCDR with all omics data available in GDSC. We used the DeepCDR implementation available at: https://github.com/kimmo1019/DeepCDR.

The pan-drug models were evaluated using as input drug features and omics data (transcriptomics for KNN, baseline NN, and PaccMann; genomics, transcriptomics, and epigenomics for DeepCDR) or drug features with zero-filled omics feature vectors. For both above cases, the models predicted either actual or z-scored IC_50_ values, obtained by subtracting from each observed value the mean and dividing by the standard deviation, as computed across all cell lines/organoids on that drug. Overall, this resulted in a total of 4 test settings (matrix legend in Fig. 1c), namely IC_50_ with original omics (blue), IC_50_ with zero-filled omics (light orange), z-scored IC_50_ with original omics (green), z-scored IC_50_ with zero-filled omics (dark orange).

#### Single-drug models

Single-drug models were trained separately for each drug; each single-drug model was blind to drug response of cell lines or organoids to other drugs. As a baseline for single-drug models, we tested a simplistic mean baseline model that computes, for each drug, a mean value of drug responses in the train set and uses this mean value as a prediction for all test samples. The mean baseline model only uses as input drug information and is blind to omics data. We then tested a total of 8 standard regression models, namely: k-Nearest Neighbors (KNN), Linear Regression (Ridge with regularization parameter alpha=10), Support Vector Regression (with a linear, radial basis function, and polynomial kernel), Decision Tree, Random Forest, Multi-layer Perceptron. For linear models (Linear Regression, linear Support Vector Regression), linearly independent genes were selected, so that no two genes are linearly correlated with Pearson’s R correlation coefficient smaller than 0.6 and a p-value smaller than 0.05. This was done by iteratively performing agglomerative clustering based on 1-Pearson-distances (distance threshold between clusters = 0.4, complete linkage), selecting one representative gene from each cluster, and repeating while the clusters contain more than one element. The best results were obtained with a linear regression model with genes selected using Pearson’s correlation relevance score (on average 770 genes selected per drug), which is what we report in the manuscript (Fig. 1c). The linear regression model was trained and tested with omics features only. We used a standard 5-fold cross validation scheme by splitting the dataset into 5 equal sized folds so that the folds had no overlapping cell lines. All models were implemented using scikit-learn (https://scikit-learn.org).

### Drug sensitivity prediction from PCPL

For the prediction of AUC per drug for the PCPL organoids, we used: (i) single-drug models (linear regression with selected features using Pearson’s correlation relevance score) trained on PCPL only, (ii) pan-drug models trained on GDSC and tested on PCPL, and (iii) pan-drug models trained and tested on PCPL only. DeepCDR was excluded from the evaluation, as it requires genomic features that are not available for PCPL. Pan-drug models were applied to predict response to 14 drugs present both in GDSC and PCPL, whereas single-drug models predicted response to all 26 PCPL drugs. We used a 10-fold cross validation to evaluate the PCPL models. For the last set of experiments, where the pan-drug models were trained on GDSC cell lines and tested on PCPL organoids, no cross-validation was performed; instead, the full GDSC dataset was used solely for training, while the PCPL dataset was used for testing. To make RNAseq data comparable across the two datasets (GDSC and PCPL), we z-scored each dataset separately and then z-scored the merged dataset one more time.

### Gene selection

To quantify the dependence of each gene in the gene expression data to each drug, we computed a set of the following 5 scores between gene expression level and drug response across all cell lines or organoids screened with that drug: (i) Pearson’s correlation coefficient and (ii) Spearman’s R correlation coefficient, (iii) mutual information score^35^, (iv) sum of squared residuals of the least squares polynomial fit of the second degree, (v) coefficients of linear SVM regression predicting drug response based on gene expression level. We then used 100 thresholds on each relevance score for filtering out irrelevant genes with the goal to determine which combination of score, threshold, and model provides the best performance for each drug based on average Pearson’s correlations between observed and predicted drug response per cell line/organoid. The experiments were conducted in a cross-validation setting.

### Performance assessment scores

To evaluate the performance of all models we used four following scores:

(i) the Pearson correlation coefficient between observed and predicted drug response measures, as computed separately per patient and per drug:
(ii) the mean squared error (MSE) of the prediction as computed separately per patient and per drug.
(iii) Precision at *k*, i.e., the percentage of correct predictions of top-*k* drugs being ranked according to the selected scores (IC_50_, IC_50_ z-score, AUC, AUC z-score).
(iv) the normalized discounted cumulative gain at *k*, i.e. the sum of relevance values of items occupying the first *k* ranks normalized by the measure of the ideal ground truth ranking, for more details see (Prasse et al.)^18^.

### Reporting summary

Further information on research design is available in the Nature Research Reporting Summary linked to this article.

### Supplementary information

Supplementary material

Supplementary Data 1

Supplementary Data 2

Supplementary Data 3

Supplementary Data 4

Supplementary Data 5

Supplementary Data 6

REPORTING SUMMARY

## Data Availability

In this study we used the following datasets: (i) GDSC1, available from the Genomics of Drug Sensitivity in Cancer portal (https://www.cancerrxgene.org/downloads/drug_data?screening_set=GDSC1); drug pathways were downloaded from the same link under Preview: drugs included in download (.csv), (ii) CCLE, available from DepMap (https://depmap.org/portal/download/all/), (iii) CTRP (v2), available from Rees et al.^23^ as Supplementary files (Supplementary Datasets 1–3), and (iv) PCPL, available from Tiriac et al.^19^ (Supplementary Table S4).

## Code availability

The code related to this study, including figure-generating scripts, is available under an open-source license at: https://github.com/Urogenus/GDSC_Pancreatic_study.
