# Supplementary material for: Overcoming limitations in current measures of drug response may enable AI-driven precision oncology
Source: NPJ Precis Oncol. 2024 Apr 24;8:95. doi: 10.1038/s41698-024-00583-0 (PMC11043358; doi:10.1038/s41698-024-00583-0)
Supplement: Supplementary file 1 — Supplementary material [file 41698_2024_583_MOESM1_ESM.pdf]

Supplementary Material: Overcoming limitations in current measures of drug response may enable AI-driven precision oncology

Katja Ovchinnikova[1], Jannis Born[2], Panagiotis Chouvardas[3], Marianna Rapsomaniki[2,4*] and Marianna Kruithof-de Julio[1,3*]

[1] *Urology Research Laboratory, Department for BioMedical Research, University of Bern, Bern, Switzerland*
[2] *IBM Research Europe, Zurich, Switzerland*
[3] *Department of Urology, Inselspital, Bern University Hospital, University of Bern, Bern, Switzerland*
[4] *Current address: Biomedical Data Science Center, Lausanne University Hospital, Lausanne, Switzerland*
[*] *Co-corresponding authors. E-mails: MAR: marianna.rapsomaniki@unil.ch , MKdJ: marianna.kruithofdejulio@unibe.ch*

Supplementary Figures

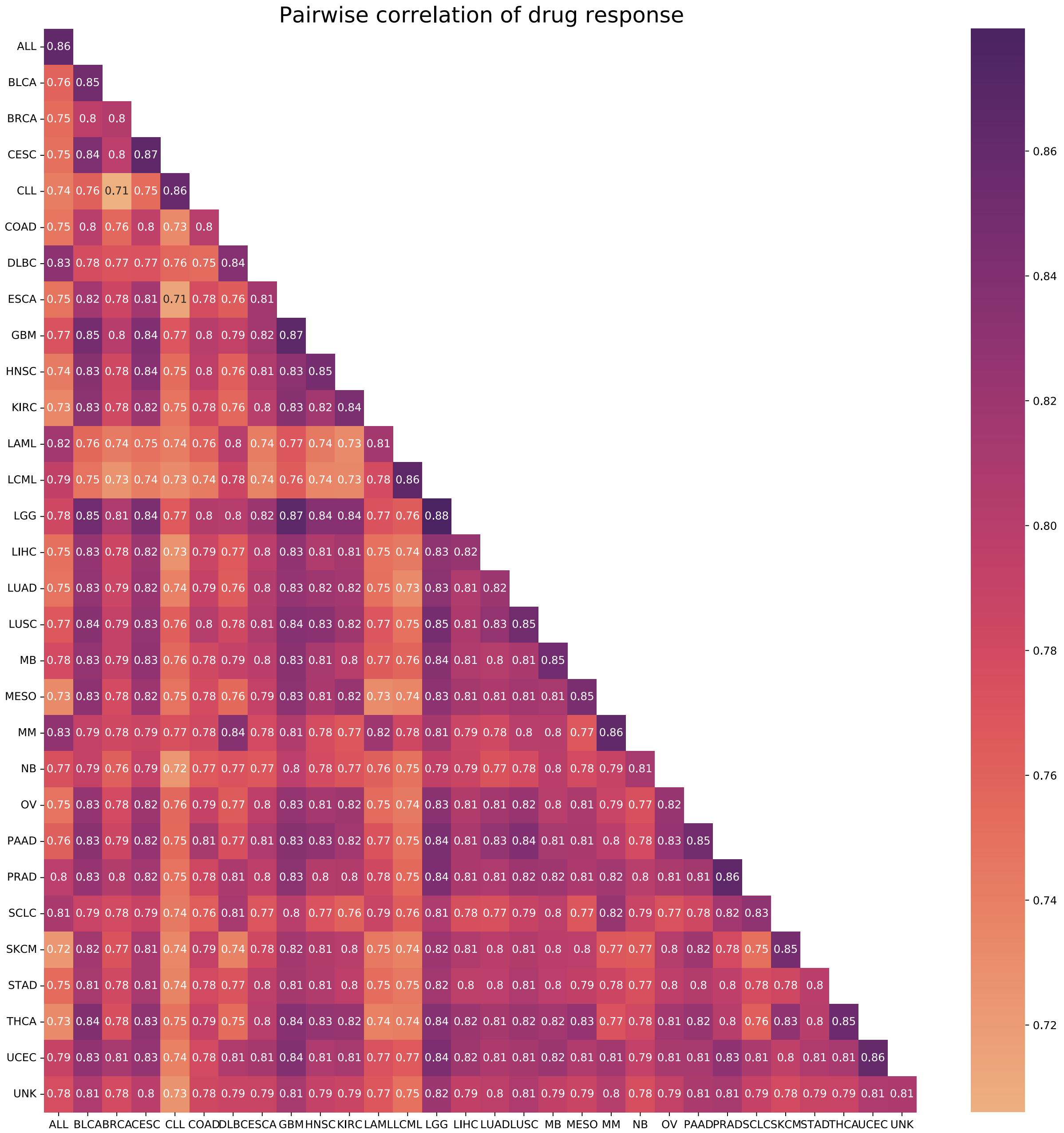

Supplementary Figure 1: Heatmap of pairwise Pearson's correlation coefficients between cell lines belonging to different cancer types from the GDSC database indicates that drug response prediction, as measured by IC50, is strongly correlated even between cell lines of distinct origins (minimum observed correlation coefficient = 0.71). The phenomenon is more pronounced between cell lines belonging to the same cancer types, as values on the diagonal have a minimum correlation coefficient of 0.8.

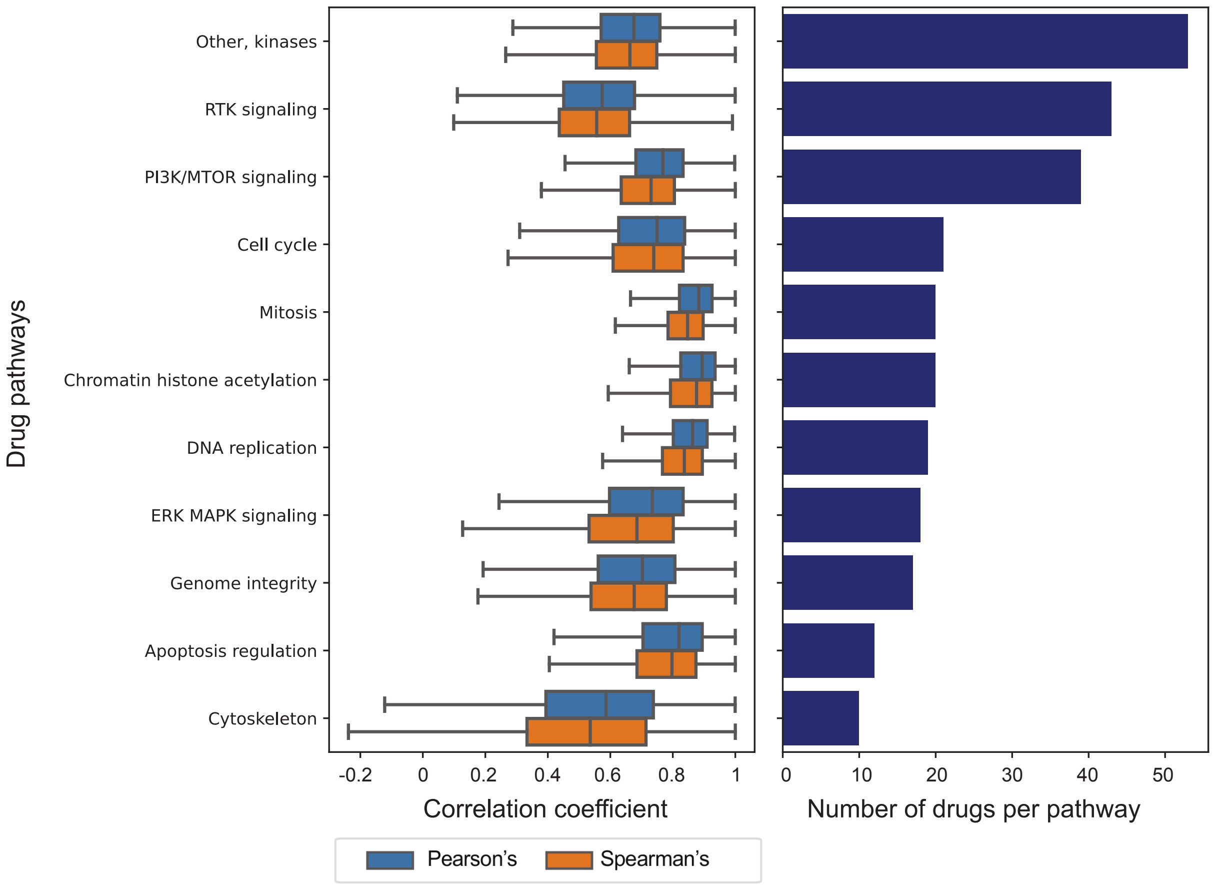

Supplementary Figure 2: Boxplots of pairwise Pearson and Spearman correlation coefficients computed for drugs belonging to different pathways from the GDSC database (left). Number of drugs per pathway (right). Pathways were selected if the included at least 10 drugs and were tested in at least 5 cell lines; pathways marked as "Unclassified" or "Other" were excluded from the analysis.

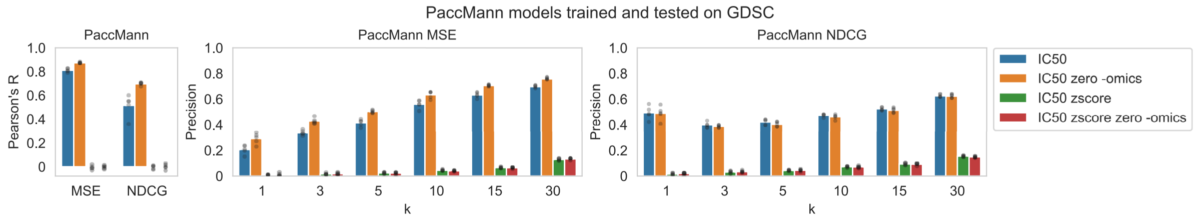

Supplementary Figure 3: Evaluation of PaccMann models using Pearson's R correlation coefficient (left), Mean Squared Error – MSE (middle) and a normalized discounted cumulative gain – NDCG (right) as a loss function. All PaccMann models were trained and tested on GDSC for four different test settings as indicated in the legend.

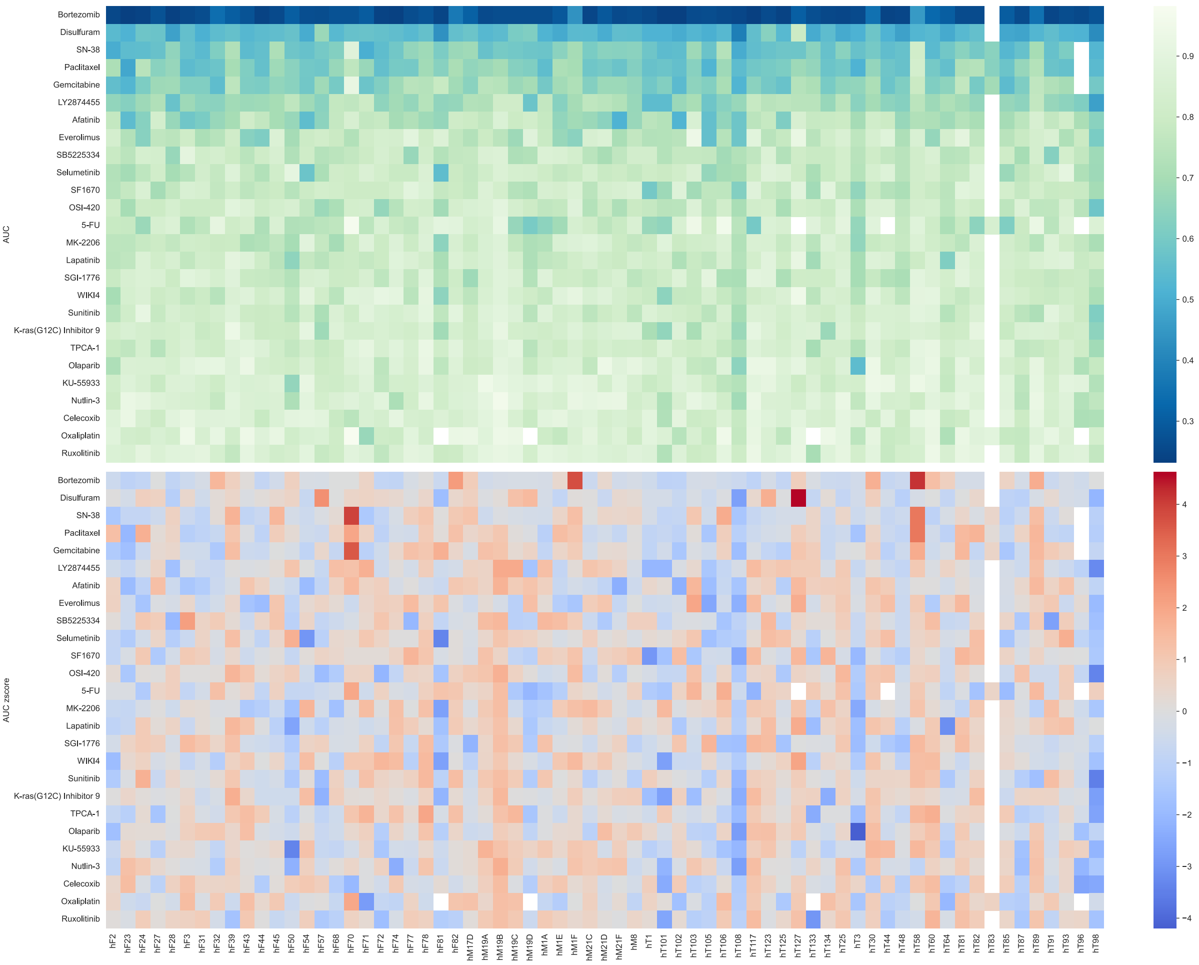

Supplementary Figure 4: Heatmap of AUC scores (top) and z-scored AUC scores (bottom) values for all PCPL organoids (columns) and all drugs tested (row).

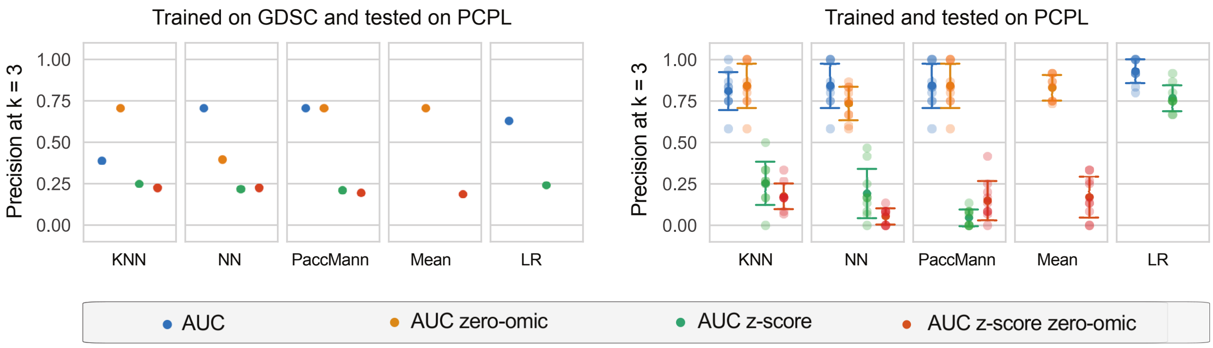

Supplementary Figure 5: Precision at k = 3 for predicted and observed drug response values computed per organoid for each of the evaluated models. All models were either trained on GDSC and tested on the PCPL dataset (left) or trained and tested on the PCPL dataset only (right). In the latter, error bars indicate the standard deviation across 10 cross validation folds.

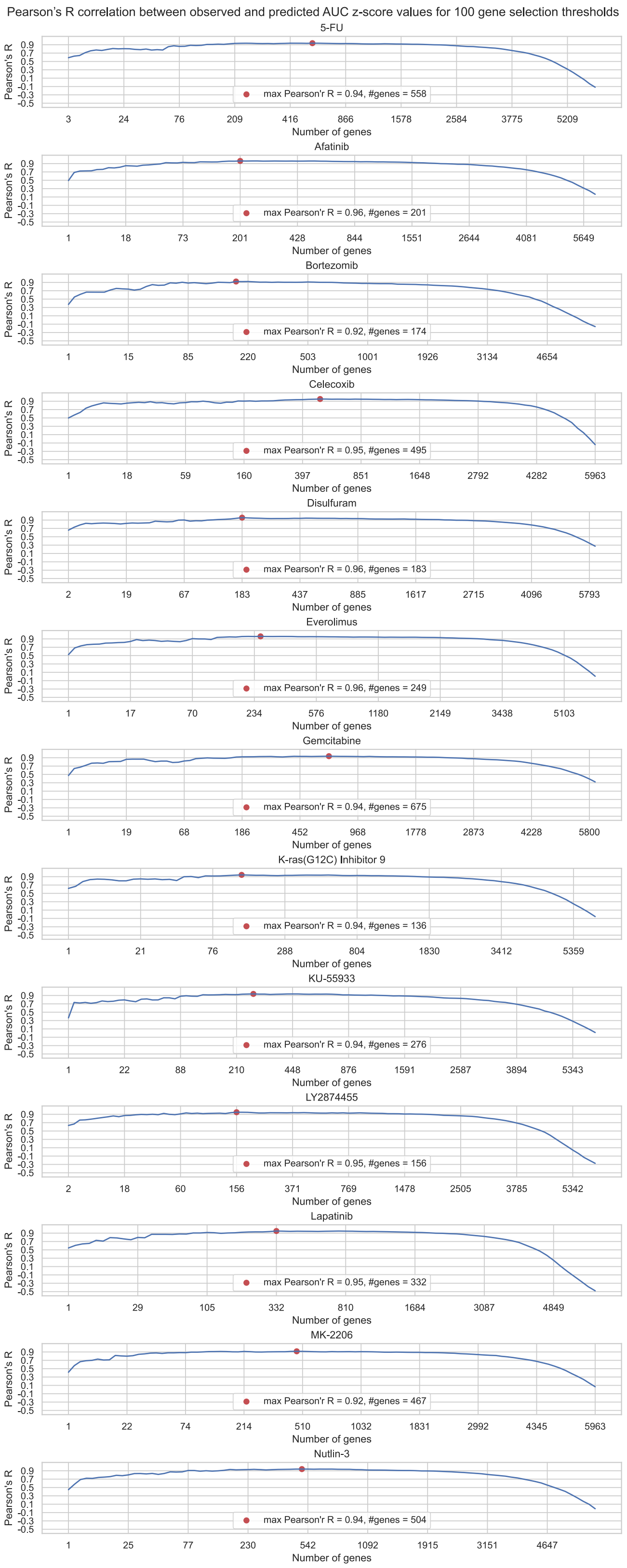

Supplementary Figure 6: Pearson's R correlation coefficient between observed and predicted AUC z-score values for 100 gene selection thresholds across all drugs.

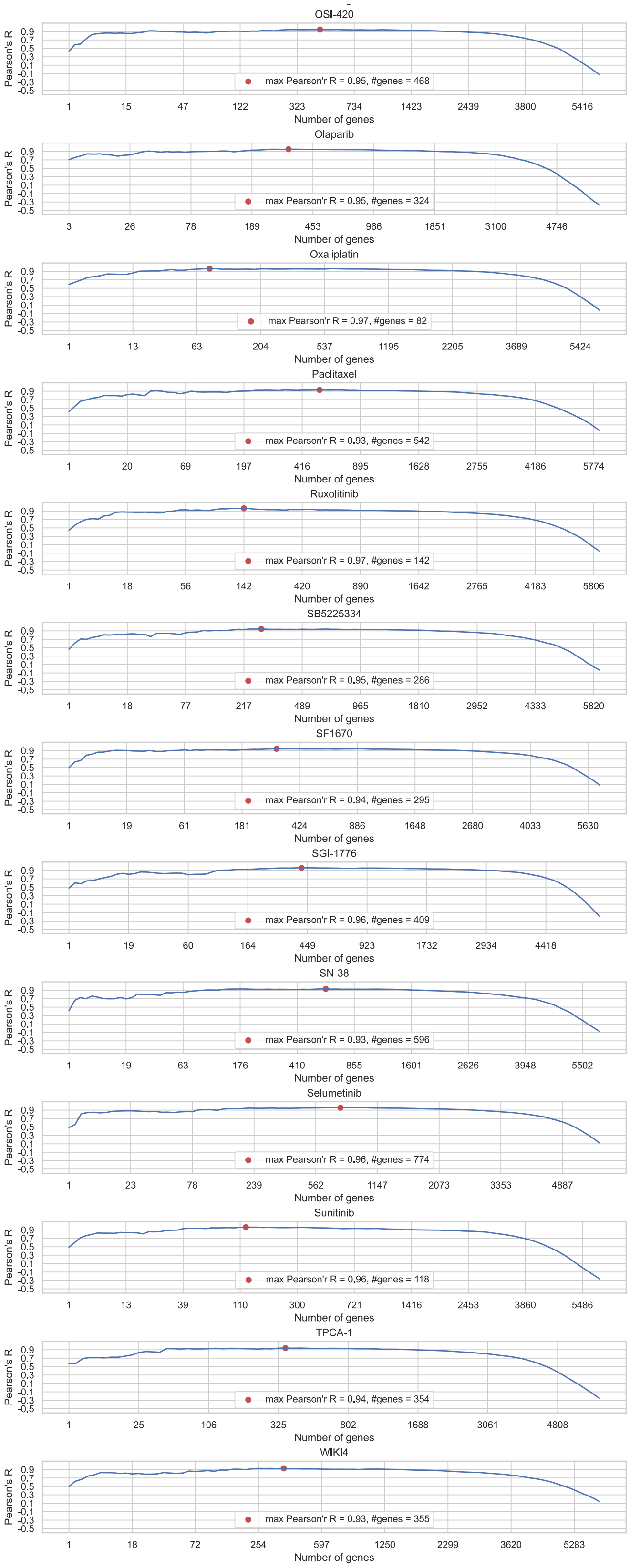

Supplementary Figure 6: *Continued.* Pearson's R correlation coefficient between observed and predicted AUC z-score values for 100 gene selection thresholds across all drugs.

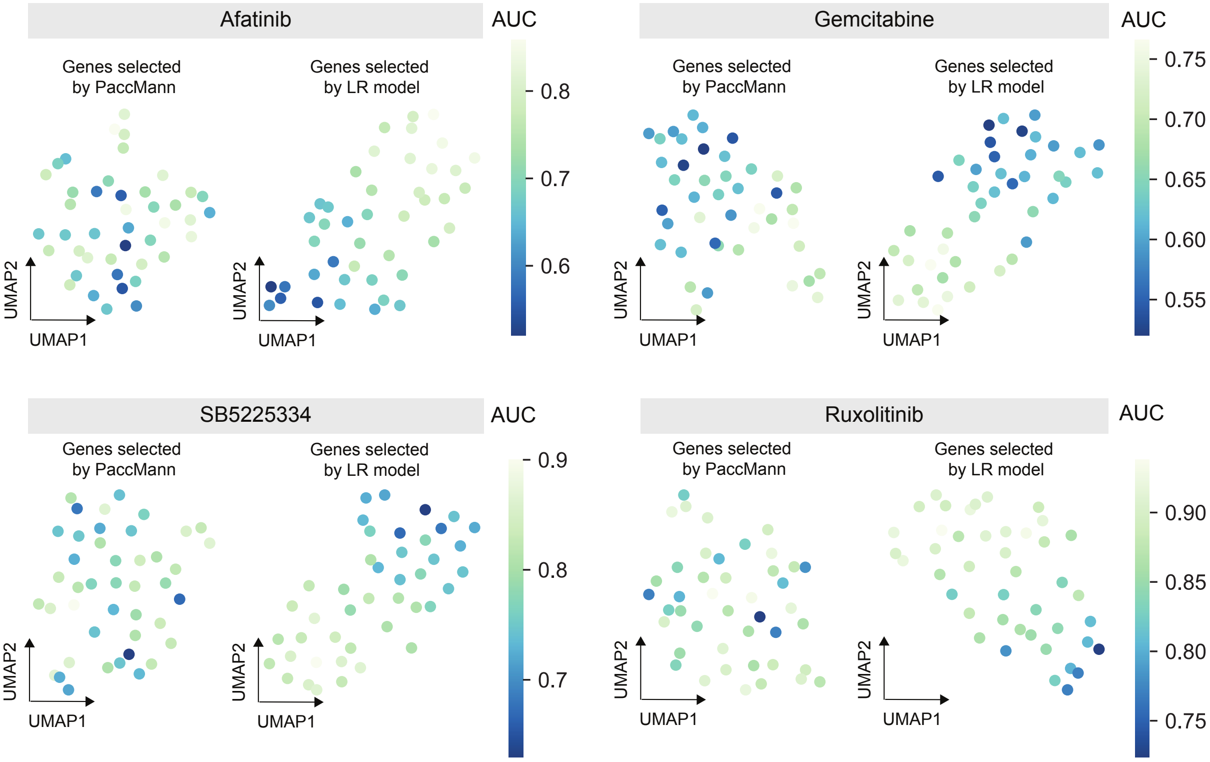

Supplementary Figure 7: UMAP embeddings of the RNAseq organoid data using genes selected by PaccMann (left) and the drug-specific gene selection scheme of the linear regression model (right) for four different drugs. Here, each dot corresponds to an organoid, and the color of the dot reveals the AUC values of each drug on that organoid.

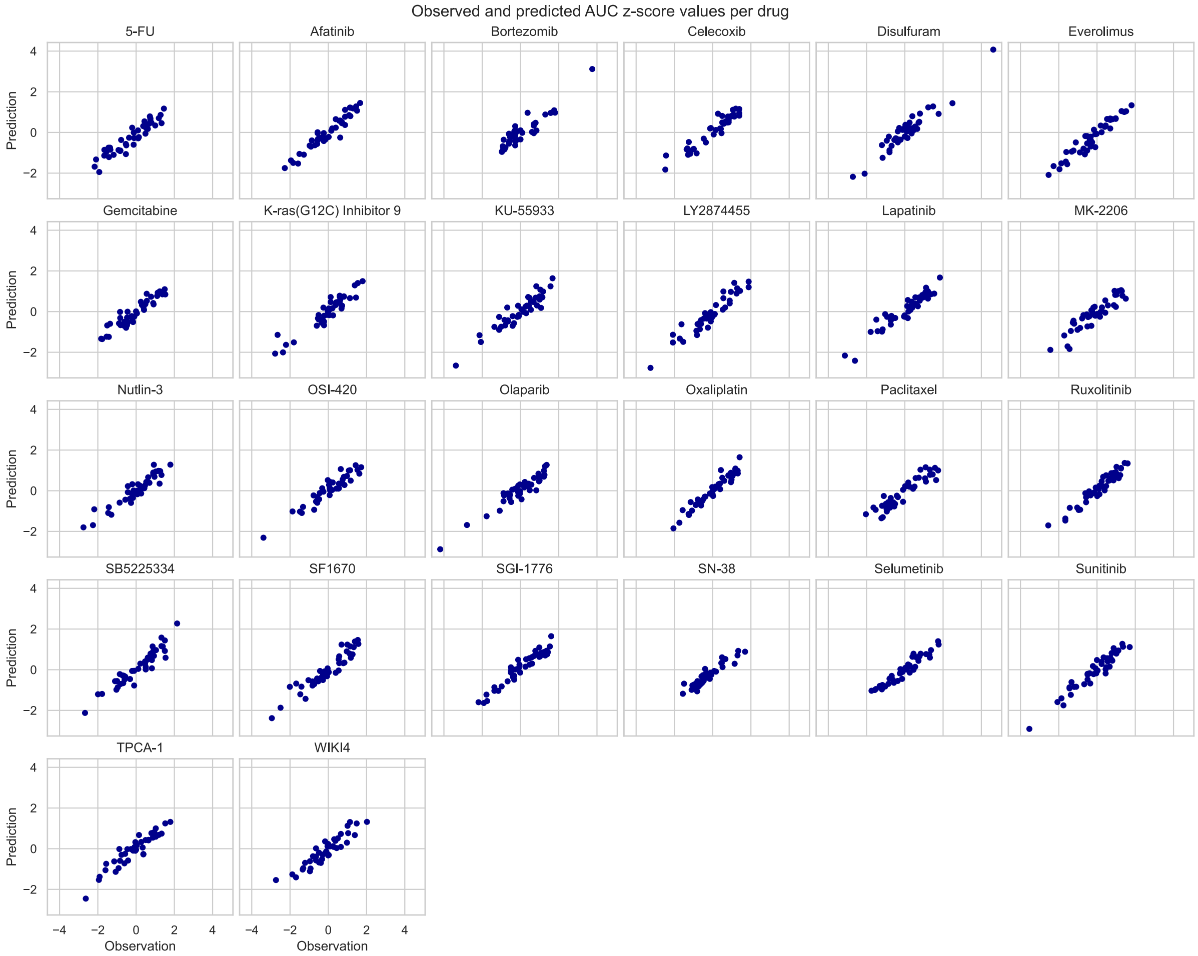

Supplementary Figure 8: Observed and predicted AUC z-score values per drug across all PCPL organoids for the drug-specific linear regression models.

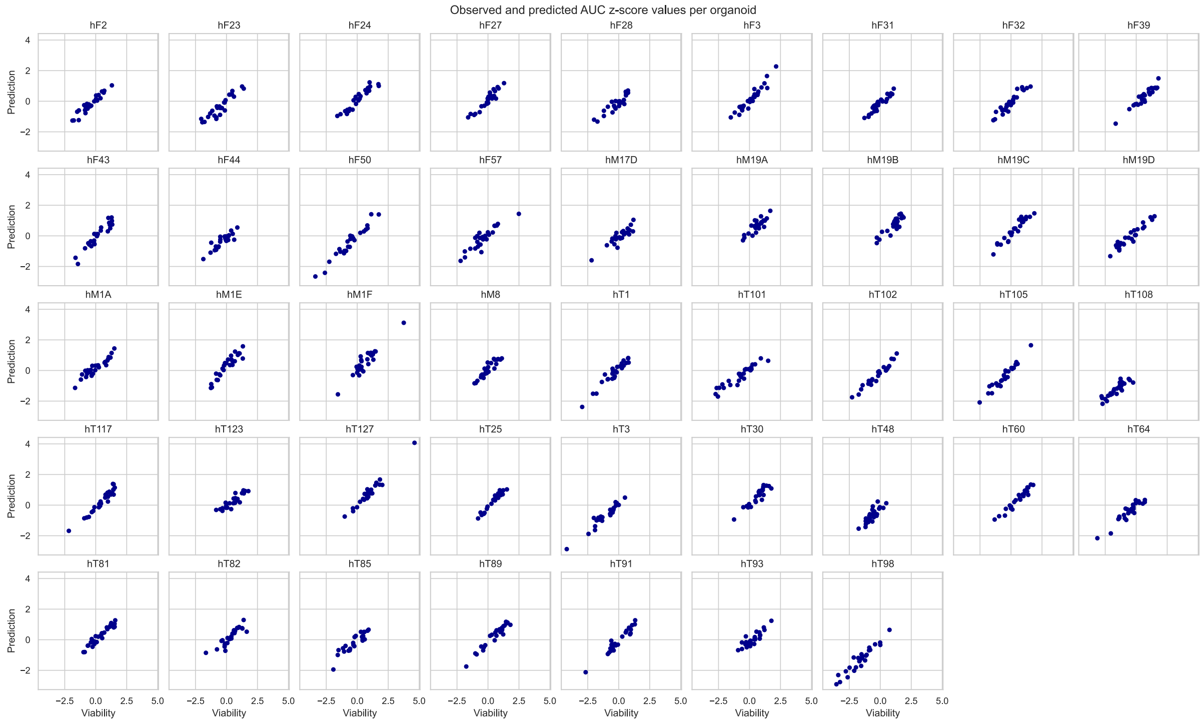

Supplementary Figure 9: Observed and predicted AUC z-score values per PCPL organoid across all drugs for the drug-specific linear regression models.

Supplementary Data Legends

Supplementary Data 1. Average Pearson's R and MSE per cell line for each model evaluated on GDSC dataset with std and std error over 5 folds.

Supplementary Data 2. Average precision at k = 1, 3, 5, 10, 15, 30 per cell line for each model evaluated on GDSC dataset with std and std error over 5 folds.

Supplementary Data 3. Average Pearson's R and MSE per drug for each model evaluated on GDSC dataset with std and std error over 5 folds.

Supplementary Data 4. Average Pearson's R and MSE per organoid for each model evaluated on PCPL dataset with std and std error over 10 folds.

Supplementary Data 5. Average precision at k = 1, 3, 5, 10, 15 per organoid for each model evaluated on PCPL dataset with std and std error over 5 folds.

Supplementary Data 6. Average Pearson's R and MSE per drug for each model evaluated on PCPL dataset with std and std error over 10 folds.
