# Supplementary material for: Overcoming limitations in current measures of drug response may enable AI-driven precision oncology
Source: NPJ Precis Oncol. 2024 Apr 24;8:95. doi: 10.1038/s41698-024-00583-0 (PMC11043358; doi:10.1038/s41698-024-00583-0)
Supplement: Supplementary file 8 — REPORTING SUMMARY [file 41698_2024_583_MOESM8_ESM.pdf]

nature portfolio

Corresponding author(s): Maria Anna Rapsomaniki, Marianna Kruithof-de Julio

Last updated by author(s): Jan 15, 2024

# Reporting Summary

Nature Portfolio wishes to improve the reproducibility of the work that we publish. This form provides structure for consistency and transparency in reporting. For further information on Nature Portfolio policies, see our Editorial Policies and the Editorial Policy Checklist.

## Statistics

For all statistical analyses, confirm that the following items are present in the figure legend, table legend, main text, or Methods section.

| n/a | Confirmed | |
|---|---|---|
| ☒ | ☐ | The exact sample size ($n$) for each experimental group/condition, given as a discrete number and unit of measurement |
| ☒ | ☐ | A statement on whether measurements were taken from distinct samples or whether the same sample was measured repeatedly |
| ☒ | ☐ | The statistical test(s) used AND whether they are one- or two-sided<br>*Only common tests should be described solely by name; describe more complex techniques in the Methods section.* |
| ☒ | ☐ | A description of all covariates tested |
| ☒ | ☐ | A description of any assumptions or corrections, such as tests of normality and adjustment for multiple comparisons |
| ☐ | ☒ | A full description of the statistical parameters including central tendency (e.g. means) or other basic estimates (e.g. regression coefficient) AND variation (e.g. standard deviation) or associated estimates of uncertainty (e.g. confidence intervals) |
| ☒ | ☐ | For null hypothesis testing, the test statistic (e.g. $F$, $t$, $r$) with confidence intervals, effect sizes, degrees of freedom and $P$ value noted<br>*Give P values as exact values whenever suitable.* |
| ☒ | ☐ | For Bayesian analysis, information on the choice of priors and Markov chain Monte Carlo settings |
| ☐ | ☒ | For hierarchical and complex designs, identification of the appropriate level for tests and full reporting of outcomes |
| ☐ | ☒ | Estimates of effect sizes (e.g. Cohen's $d$, Pearson's $r$), indicating how they were calculated |

*Our web collection on statistics for biologists contains articles on many of the points above.*

## Software and code

Policy information about availability of computer code

| | |
|---|---|
| Data collection | All code related to this study is available under an open-source license at: https://github.com/Urogenus/GDSC_Pancreatic_study |
| Data analysis | All code related to this study is available under an open-source license at: https://github.com/Urogenus/GDSC_Pancreatic_study. All models were implemented using scikit-learn (https://scikit-learn.org). More advanced machine learning models were tested using their original implementations:<br>- PaccMann implementation from https://github.com/PaccMann/paccmann_predictor.<br>- DeepCDR implementation available at: https://github.com/kimmo1019/DeepCDR. |

For manuscripts utilizing custom algorithms or software that are central to the research but not yet described in published literature, software must be made available to editors and reviewers. We strongly encourage code deposition in a community repository (e.g. GitHub). See the Nature Portfolio guidelines for submitting code & software for further information.

## Data

Policy information about availability of data

All manuscripts must include a data availability statement. This statement should provide the following information, where applicable:

- Accession codes, unique identifiers, or web links for publicly available datasets
- A description of any restrictions on data availability
- For clinical datasets or third party data, please ensure that the statement adheres to our policy

In this study we used the following publicly available datasets: (i) GDSC1, available from the Genomics of Drug Sensitivity in Cancer portal (https://www.cancerrxgene.org/downloads/drug_data?screening_set=GDSC1); drug pathways were downloaded from the same link under Preview: drugs included in download (.csv), (ii) CCLE, available from DepMap (https://depmap.org/portal/download/all/), (iii) CTRP (v2), available from Rees et al.23 as Supplementary files (Supplementary Datasets 1-3), and (iv) PCPL, available from Tiriac et al.19 (Supplementary Table S4).

## Research involving human participants, their data, or biological material

Policy information about studies with human participants or human data. See also policy information about sex, gender (identity/presentation), and sexual orientation and race, ethnicity and racism.

| | |
|---|---|
| Reporting on sex and gender | This study did not recruit any patients or generate any new data, but rather re-used already available public in vitro or ex vivo datasets (see above). For the PCPL dataset, information on gender is available at Table S1 from Tiiac et al., 2018. |
| Reporting on race, ethnicity, or other socially relevant groupings | n/a |
| Population characteristics | n/a |
| Recruitment | n/a - We  generate any new data in this study. |
| Ethics oversight | n/a |

Note that full information on the approval of the study protocol must also be provided in the manuscript.

# Field-specific reporting

Please select the one below that is the best fit for your research. If you are not sure, read the appropriate sections before making your selection.

☒ Life sciences ☐ Behavioural & social sciences ☐ Ecological, evolutionary & environmental sciences

For a reference copy of the document with all sections, see nature.com/documents/nr-reporting-summary-flat.pdf

# Life sciences study design

All studies must disclose on these points even when the disclosure is negative.

| | |
|---|---|
| Sample size | For the analysis shown in Figure 1, we used all available data from the GDSC, CCLE, CTRP and PCPL datasets. |
| Data exclusions | We did not exclude any data from the main analyses. For Supplementary Fig.S2, we excluded pathways with less than 10 drugs tested in less 5 cell lines. |
| Replication | For all ML models, we ran five independent cross-validation folds, and report the corresponding results and standard deviation in Fig.1A for GDSC and Fig.2C for PCPL. |
| Randomization | In the context of training the ML models, dataset division in training and test splits was performed randomly |
| Blinding | n/a |

# Reporting for specific materials, systems and methods

We require information from authors about some types of materials, experimental systems and methods used in many studies. Here, indicate whether each material, system or method listed is relevant to your study. If you are not sure if a list item applies to your research, read the appropriate section before selecting a response.

## Materials & experimental systems

| n/a | Involved in the study |
|---|---|
| ☒ | ☐ Antibodies |
| ☒ | ☐ Eukaryotic cell lines |
| ☒ | ☐ Palaeontology and archaeology |
| ☒ | ☐ Animals and other organisms |
| ☒ | ☐ Clinical data |
| ☒ | ☐ Dual use research of concern |
| ☒ | ☐ Plants |

## Methods

| n/a | Involved in the study |
|---|---|
| ☒ | ☐ ChIP-seq |
| ☒ | ☐ Flow cytometry |
| ☒ | ☐ MRI-based neuroimaging |

# Plants

| | |
|---|---|
| Seed stocks | n/a |
| Novel plant genotypes | n/a |
| Authentication | n/a |
